# Retraction: Unlocking employee creativity: How learning orientation and transformational leadership spark innovation through creative self-efficacy

**DOI:** 10.1371/journal.pone.0352735

**Published:** 2026-07-08

**Authors:** The PLOS One Editors

The *PLOS One* Editors retract this article [1] due to concerns about:

- Extensive text similarity with potential paraphrasing, and high similarity in study design, sample, results, and conclusions with a study previously reported in [2], cited in [1] as Reference 27.
- Compliance with PLOS policy on Authorship and adherence to the journal’s third publication criterion [3].
- The articles cited as References 8 and 44 were retracted before publication of [1].
- Anomalies in the underlying dataset, including values that do not align with the described methods and results.

The second, third, and fourth authors disagreed with the concern regarding similarity between the two articles [1,2], stating that [2] investigates transformational leadership as a single construct, while [1] investigates transformational leadership separated into four sub-dimensions. In comparing the methods of the article, the Editors note that the same questionnaire related to the four sub-dimensions was used to assess transformational leadership in both studies.

The authors also stated that the studies surveyed different samples at different times. Both articles report the sample as representing small and medium-sized enterprises in China’s manufacturing sector, but lack information on the source of the sample population and how individuals were recruited. The lack of recruitment information raises concerns about reliability and reproducibility of [1], and the authors’ responses do not resolve the concerns regarding the use of a similar sample in both studies.

The *PLOS One* Editors do not consider that these responses resolve the concerns identified.

All authors did not agree with the retraction.
